# Facial soft-tissue shape changes after fixed edgewise treatment with premolar extraction in individual artificial-intelligence-classified facial profile patterns

**DOI:** 10.1186/s12903-024-04512-2

**Published:** 2024-06-27

**Authors:** Chihiro Tanikawa, Tzee Jen Tan, Kenji Takada

**Affiliations:** 1 Department of Orthodontics and Dentofacial Orthopedics, Osaka University Dental Hospital, 1-8, Yamadaoka, Suita, 565-0871 Osaka Japan; 2 Center for Advanced Medical Engineering and Informatics, Osaka University, Suita, Osaka Japan; 3 Faculty of Dentistry, National University of Singapore, Singapore, Republic of Singapore

**Keywords:** Humans, Lip, Artificial intelligence, Tooth extraction, Esthetics

## Abstract

**Objective:**

To examine the patterns of pretreatment facial soft tissue shape in orthodontic cases with premolar extraction using artificial intelligence (AI) and to investigate the corresponding changes.

**Methods:**

One hundred and fifty-two patients who underwent orthodontic treatment with premolar extraction were enrolled. Lateral cephalograms were obtained before and after the treatment. For each record, the outlines of the nose-lip-chin profile and corresponding 21 cephalometric variables were extracted. The AI method classified pretreatment records into three subject groups based on the feature variables extracted from the outline. Dentoskeletal and soft tissue facial form changes observed after treatment were compared statistically (*P* < 0.05) between the groups using ANOVA. Multivariate regression models were used for each group.

**Results:**

Group 1 (*n* = 59) was characterized by Class II high-angle retrognathic mandible with an incompetent lip, group 2 (*n* = 55) by Class I malocclusion with retruded and thin lips, and group 3 (*n* = 38) by Class I malocclusion with an everted superior lip before treatment. The ratios of anteroposterior soft tissue to hard tissue movements in Group 1 were 56% (*r* = 0.64) and 83% (*r* = 0.75) for the superior and inferior lips, respectively, whereas those in Group 2 were 49% (*r* = 0.78) and 91% (*r* = 0.80), and 40% (*r* = 0.54) and 79% (*r* = 0.70), respectively, in Group 3.

**Conclusions:**

The modes of facial form changes differed depending on the pre-treatment profile patterns classified by the AI. This indicates that the determination of the pre-treatment profile pattern can help in the selection of soft tissue to hard tissue movement ratios, which helps estimate the post-treatment facial profile with a moderate to high correlation.

**Supplementary Information:**

The online version contains supplementary material available at 10.1186/s12903-024-04512-2.

## Introduction

In orthodontics, facial attractiveness is the primary motivation for patients seeking treatment [1]. Bimaxillary protrusion is characterized by proclined maxillary and mandibular incisors, and increased procumbency of the lips [2]. Asians show a greater tendency toward bilabial protrusion than other populations [3, 4], which often causes problems such as difficulty in closing the mouth or lip incompetency. Addressing both aesthetic and functional issues is an important goal of orthodontic treatment; therefore, premolar tooth extraction is often the first choice for management. Examining the relationship between hard and soft tissues and estimating facial morphology after orthodontic treatment with tooth extraction is crucial for orthodontists and patients.

There have been many reports on the improvement in morphology after orthodontic treatment with tooth extraction. Most studies have examined the differences between extraction and non-extraction group [5–9] or the correlation coefficients between the soft tissue components of the superior and inferior lips and hard tissue [10–13]. The ratio between the soft tissue component of the superior and inferior lips and the hard tissue varies broadly among cases, and the correlation coefficient values have only shown a weak to medium correlation, especially in the inferior [14] or superior lips [13]. Nevertheless, despite the difficulty in determining consistent values for the ratio, such values have been used clinically to predict facial soft tissue patterns, and patients decide on a treatment method based on this prediction.

The past two decades have seen the development and spread of artificial intelligence (AI). The core of AI is machine learning, which includes a classifier that can create typical patterns (usually called training) based on the numbers extracted from targeted objects such as images (usually called feature variables). Classification is generally performed such that the algorithm minimizes within-group variation while maximizing between-group differences. That is, AI-induced classification of patients may reduce the variation among the population by grouping similar subjects, which minimizes the variation in each patient group [15] and helps in understanding the relationship between the hard and soft tissues in each group.

Therefore, the present study aimed to cluster the facial profiles of a group of patients who underwent orthodontic treatment with tooth extraction using an AI algorithm and then to investigate the relationship between changes in the incisor positions and facial profiles within the determined groups.

## Materials and methods

### Data collection

This retrospective cohort study examined a series of consecutive patients who underwent edgewise treatment with four premolar extractions and visited two private clinics in Japan and Singapore between 2008 and 2012. The inclusion criteria were as follows: (1) East Asian women aged 12–39 years, (2) patients with skeletal Class I or II malocclusions with an ANB angle > 0, (3) the eruption of all second molars, (4) Completion of post- orthodontic treatment, and (5) pre-and post-treatment cephalometric radiographs. Patients with craniofacial anomalies, missing teeth (except third molars), severe mandibular deviation > 5 mm, obesity, a history of facial trauma, a history of maxillofacial plastic surgery, and incomplete diagnostic records were excluded. As a result, 152 patients (age range:12.0-37.5 years old) were enrolled. Lateral cephalograms were obtained before and after edgewise treatment.

All experimental protocols were approved by the Research Ethics Committee of Osaka University Dental Hospital (No. H25-E6). Informed consent was obtained from all patients and/or their legal guardians using the opt-out method.

### Processing of cephalometric values

First, each film was traced with a pencil on an acetate paper overlaid on the films by one author (CT) and double-checked by another author (KT). Films with traced papers were digitized using a scanner (ES8500; EPSON, Tokyo, Japan) at a resolution of 300 dots per inch to provide a traced image dataset (2,320 × 2,960 pixels, 1 pixel = 0.085 mm).

Second, each traced image was digitized using a computer mouse and one of the authors (CT) identified the anatomical landmarks for the traced image data on a computer monitor (24-inch pen tablet monitor, 1,920 × 1,200 dots, Cintiq 24HD; Wacom, Saitama, Japan). This process was repeated twice for all images and the landmark coordinates from both digitization processes were averaged to obtain the final landmark coordinates. Based on landmark coordinates, 21 cephalometric variables (Table 1) were measured using a customized software program. All the calculations were performed using MATLAB 2022 A (MathWorks, Natick, MA, USA).

**Table 1:** Cephalometric measurements

Measurement	Definition
SN (mm)	Distance between the Sella and the Nasion
SNA (deg.)	Angle formed by the SN line and the NA line
SNPP (deg.)	Angle formed by the SN plane and the palatal plane
A-Ptm/PP (mm)	Point A to Ptm distance projected on the palatal plane
SNB (deg.)	Angle formed by the SN line and the NA line
ANB (deg.)	Angle formed by Point A, Nasion and Point B
SNMP (deg.)	Angle formed by the SN plane and the mandibular plane (Go-Me)
Me/PP (mm)	Anterior lower facial height
Ar-Me (mm)	Length between the Articulare and Menton
U1-SN (mm)	Angle formed by the long axis of the maxillary central incisor and the SN line
L1-FH (mm)	Angle formed by the long axis of the mandibular central incisor and the Frankfort Horizontal (FH) plane
OJ (mm)	Overjet
OB (mm)	Overbite
U1-ls_x, U-1s_y (mm)*	Horizontal and vertical distances between the maxillary central incisor tip and the labial superior, respectively
L1_ls_x, L1_ls_y (mm)*	Horizontal and vertical distances, between the mandibular central incisor tip and the labial superior, respectively
U1_li_x, U1-li_y (mm)*	Horizontal and vertical distances, between the maxillary incisor tip and the labial inferior, respectively
L1_li_x, L1-Li_y (mm)*	Horizontal and vertical distances, between the mandibular central incisor tip and the labial inferior, respectively
*, For U1-ls_y, L1-ls_y, U1-li_y, and L1-li_y, smaller values indicate superior or inferior lips located in vertically higher positions than the maxillary or mandibular central incisors.	

To analyze the soft tissue profile, a tablet pen (attached to the monitor described above) was employed to trace the soft tissue profiles of the traced image data on the computer monitor, thus providing a series of coordinates for the facial profiles.

A customized software program automatically calculated 13 variables from the profile coordinates, as shown in Supplementary Fig. 1 (v1, v2, …, v13), based on the method used to classify nasolip-chin profiles in our previous study [15]. In our study, the lateral facial photographs and cephalograms of 229 Japanese women were examined before orthodontic treatment. A feature vector extracted from facial photographs (v1, v2, …, v13; Supplementary Fig. 1) was entered into an AI clustering method (vector quantization method [15]) to categorize the profiles into eight distinct patterns. The feature vector variables included the nasolabial angle, configuration, and vertical length of the subnasal region, the vertical thickness of the lip vermilion borders, sagittal position of the upper and lower lip vermilion borders, and their relationship to each other, labiodental angle, depth of the labiodental sulcus, degree of prominence of the chin, and degree of protrusion of the mandible. AI clustering allows facial profiles, each expressed by a multidimensional vector, to be categorized based on their vector similarities. The centroids of each cluster were used as mean codes (i.e., patterns), which were assumed to represent each cluster. Detailed AI clustering method was described in Supplementary Fig. 2.

In the present study, the pretreatment records were classified into three subject groups using these 13 variables and the above-mentioned AI clustering method [15]. The number of subject groups was determined using the elbow method, typically used to determine the number of clusters in a dataset. This method involves plotting the variation among the centroids of the clusters as a function of the number of clusters and selecting the elbow of the curve as the number of clusters to use. This maintains the number of clusters as large as possible with minimal overlap between clusters. In this study, to 2–8 clusters were examined, and the optimum number of subject groups was determined.

Cephalometric variables before treatment and cephalometric changes throughout treatment were compared statistically (*P* < 0.05) between groups using analysis of variance (ANOVA). Furthermore, linear regression models that estimated soft tissue changes based on hard tissue changes were developed for each subject group when the anteroposterior changes in the maxillary and mandibular central incisors were set as the independent variables, and those of the superior and inferior lips were set as the dependent variables.

## Results

**Fig. 1:**
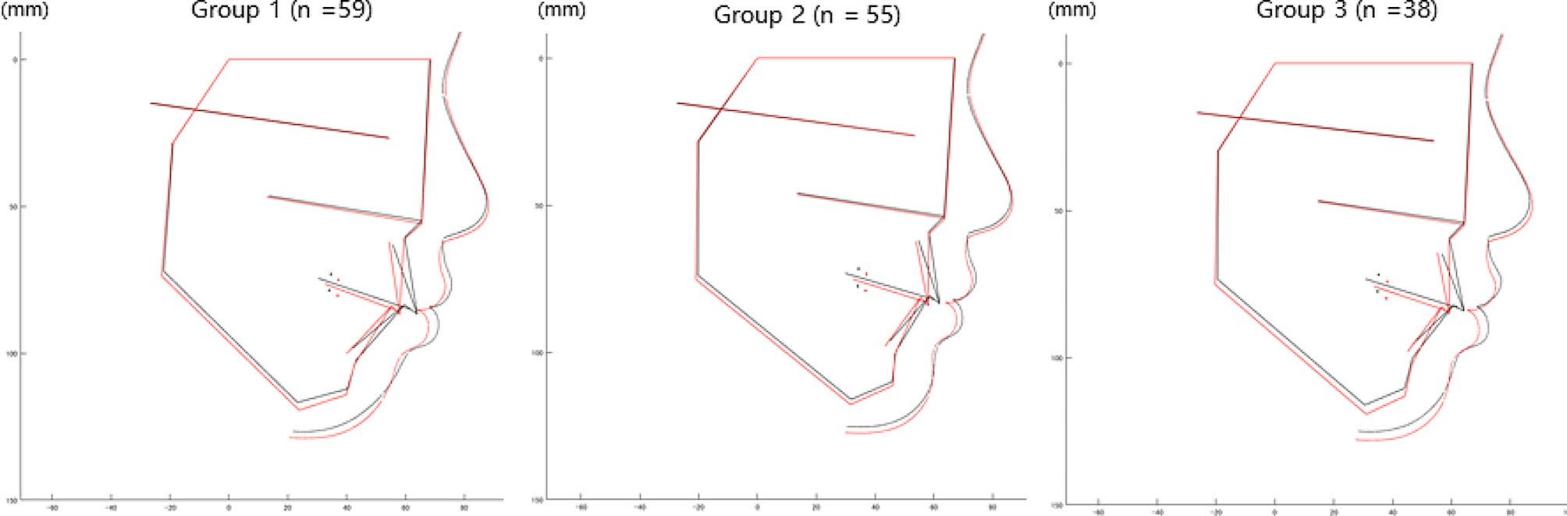
Averaged cephalometric changes for each group (pre-treatment, black line; post-treatment, red line)

The AI clustering method determined three typical subject groups for the profiles before treatment (Fig. 1): the “Class-II-high-angle group” (Group 1, *n* = 59), “Class-I-normal-angle-with-thin-lips group” (Group 2, *n* = 55), and “Class-I-with-everted-superior-lip group” (Group 3, *n* = 38). A summary of the results is shown in Table 2, and details are provided in the supplementary files (Supplementary Figs. 3 and 4). As Group 1 showed an anteroposteriorly thin superior lip, giving a convex profile of the superior lip and a shallow labiomental fold, this group was considered to have lip-incompetent characteristics.

**Table 2:** Result summary

		Group 1 (*n* = 59) “Class II high angle incompetent lip group”	Group 2 (*n* = 55) “Class I normal angle with thin lips group”	Group 3 (*n* = 38) “Class I with everted superior lip group”
Pre-tx	Skeletal	Skeletal Class II due to a smaller mandibular effective length	Class I	Class I (Class II tendency)
		High mandibular angle	Normal mandibular angle	Normal mandibular angle
		Excessive overjet		
	Soft tissue	Protruded lips	Retruded lips	Protruded lips
		Obtuse nasolabial and labiomental angles	Obtuse nasolabial angle	Acute nasolabial angle
		Convex shape of the superior lips (incompetent lip)		Concave shape of the superior lip (everted lip)
	Hard to soft distances	Antero-posteriorly thin superior lips (U1-ls_x)	Antero-posteriorly thick superior lips (U1-ls_x)	Antero-posteriorly thick superior lips (U1-ls_x)
		Lower position of the inferior lips to the maxillary central incisor (U1-li_y)	Lower position of the superior lips to the maxillary central incisors (U1-ls_y)	Higher position of the superior lips to the maxillary central incisors (U1-ls_y)
Changes following orthodontic treatment		Thickened superior lips (U1-ls_x)	Fewer changes in the thickness of the superior lips (U1-ls_x)	Vertically lengthened superior lips (U1-ls_y)
Correlation between hard- to soft-tissue responses (coefficient value r) and its inclination B	Superior lip to the maxillary incisor	Moderate (*r* = 0.64) B = 0.56	Strong (*r* = 0.78) B = 0.49	Moderate (*r* = 0.53) B = 0.40
	Inferior lip to the mandibular incisor	Strong (*r* = 0.75) B = 0.83	Strong (*r* = 0.80) B = 0.91	Strong (*r* = 0.70) B = 0.79

### Changes in hard and soft tissues following orthodontic treatment

**Fig. 2:**
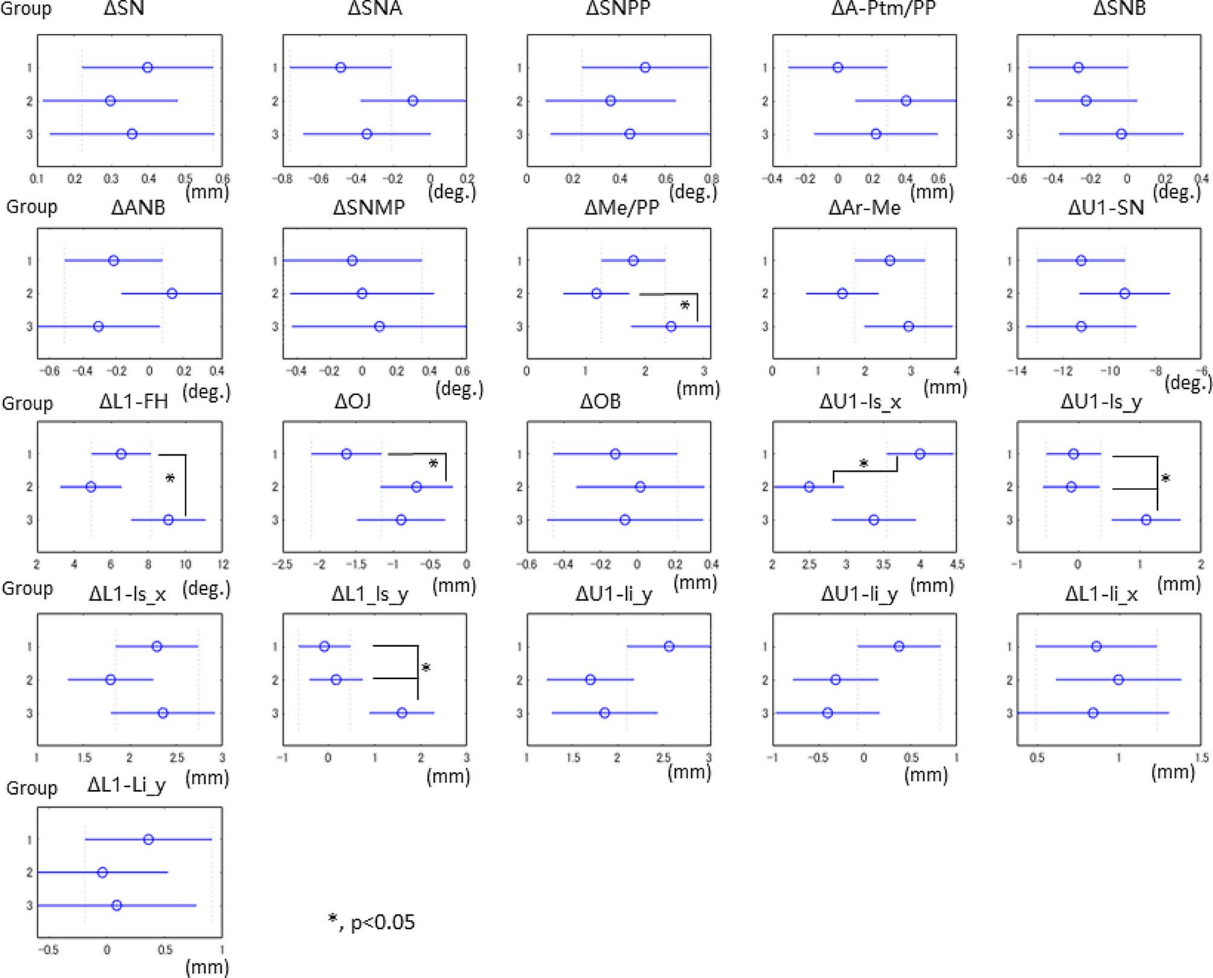
Inter-group comparisons of before and after mean changes in cephalometric measurements. *<0.05; ANOVA. Blue error bars represent Scheffé comparison intervals: nonoverlap between the bars of any two groups indicates that the hypothesis of no difference between the two sample groups was rejected at the *P* < 0.05 level. The circle points represent estimated means

A comparison of the pre- and post-treatment cephalometric records determined the posteriorly displaced maxillary and mandibular central incisors to establish proper incisor relationships in all the groups (Fig. 2). Because Group 1 showed greater overjet before treatment than Groups 2 and 3, the improvement in overjet was also the greatest among the groups.

Regarding soft tissue changes, Group 1 showed thickened superior lips following orthodontic treatment (ΔU1-ls_x). This result indicates that the superior lip in Group 1, whose superior lip was anteroposteriorly thin before treatment, thickened after treatment. Group 3 had greater downward movement of the superior lips from the maxillary and mandibular central incisors than Groups 1 and 2 (ΔU1_ls_y, ΔY1_li_y). This result indicates that the superior lip in group 3, whose superior lip was shorter before treatment, lengthened downwards after treatment.

### Correlations between the displacements of the hard and soft tissues

**Fig. 3:**
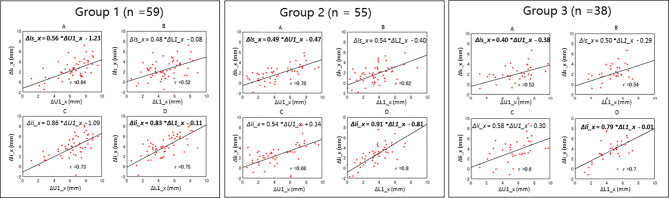
Linear regression models determined for each subject group. **A**, A scatter plot indicating a correlation between the antero-posterior changes of the maxillary central incisor (U1_x) and that of the labial superior (ls_x) during treatment; **B**, A scatter plot indicating a relationship between the antero-posterior changes of the mandibular central incisor (L1_x) and that of the labial superior (ls_x) during the treatment; **C**, A scatter plot indicating a relationship between the antero-posterior changes of the maxillary central incisor (U1_x) and that of the labial inferior (li_x) during treatment; and D, A scatter plot indicating a relationship between the antero-posterior changes of the mandibular central incisor (L1_x) and that of the labial inferior (li_x) during treatment

Figure 3 shows the linear regression models that estimated the anteroposterior changes in the maxillary and mandibular central incisors from those in the superior and inferior lips. Group 2 showed strong correlations between incisor position changes and displacement of the soft tissues in the anteroposterior direction for both the superior (*r* = 0.78) and inferior lips (*r* = 0.80). Groups 1 and 3 showed a strong correlation with the inferior lip (*r* = 0.75 and 0.70) but moderate correlations for the superior lip (*r* = 0.64 and 0.54).

The ratios of soft tissue movement to hard tissue movement in the anteroposterior direction in Group 1 were 56% and 83% for the superior and inferior lips, respectively, whereas those in Group 2 were 49% and 91%, and 40% and 79%, respectively, in Group 3.

## Discussion

In the present study, we hypothesized that AI-based classification of patients according to their profiles before orthodontic treatment could help reduce the variation in the soft tissue response to hard tissue changes. We determined three soft tissue profiles in subjects who underwent orthodontic treatment with tooth extraction using an AI algorithm. As expected, the correlation between the anteroposterior changes in the maxillary and mandibular central incisors and those in the superior and inferior lips varied among the groups. That is, the pre-treatment soft tissue status was important for examining the relationships between the hard and soft tissues in each group. This finding is consistent with those of the previous studies. For example, an AI-based three-dimensional (3D) facial prediction system showed that the shape of the face before treatment is essential for predicting the shape of the face after treatment [16]. A recent cephalometric study (*n* = 37) also showed that lip retraction at the vermilion and lip base thinning was significantly greater in patients with incompetent lips than in competent lips [17], indicating that the shape morphology before treatment can influence soft- vs. hard-tissue thickness.

Linear regression can be interpreted independently of the scale of the two variables: strength (correlation coefficient, r) and the steepness of inclination. The closer the estimated correlation is to ± 1, the closer the two are to a perfect linear relationship, indicating an accurate prediction of the soft tissue responses (or small errors from the actual facial changes). In all three groups, the inferior lip showed a strong correlation, indicating that hard tissue explains the soft tissue changes. In contrast, the response of the superior lip varied among the groups, and the “Class-I-normal-angle-with-thin-lip group” (Group 2) showed a strong correlation, but the “Class-I-with-everted-superior-lip group” (Group 3) showed a moderate correlation. While the present study showed a strong correlation in the inferior lip, a previous study showed only a weak-to-medium correlation, especially in the inferior lips [14], indicating inconsistent results. Determining the clusters before calculating the correlations in the present study may have led to strong correlations in the inferior lip.

The regression slope (inclination) is a linear relationship between two soft- and hard-tissue variables, where a greater value indicates that the high-response soft tissue changes as the hard-tissue variable changes. While the response rate for the inferior lip was as high as 80-90%, that for the superior lip was highest at 56% in the Class II high-angle group (Group 1) and lowest at 40% in the Class I with-everted-superior-lip group (Group 3). When the anteroposterior thickness of the vermilion lips was measured, the Class II high-angle group showed thin lips and incompetent morphological characteristics of the superior lip, whereas the Class I with-everted-superior-lip group showed thick lips. This result may indicate that the response of soft tissues can be influenced by lip thickness; that is, incompetent lips showed more soft tissue changes corresponding to hard tissue changes than competent lips. A previous study also showed significant variations in lip movement based on initial competency and thickness; lip retraction at the vermilion and lip-based thinning were significantly greater in patients with incompetent lips than competent lips [17]. Overall, these results show that initial competency and thickness, and thus facial profiles, should be thoroughly examined to ensure adequate profile prediction.

When the responses of the superior and inferior lips were compared, the superior lip showed a smaller response and greater variation. To explain why the responses of the superior and inferior lips differed, the following were considered: First, gravity may influence the superior lip in the vertical direction. The Class II high-angle group” showed greater downward movement of the superior lips from the maxillary and mandibular central incisors by almost 1.5 mm. This result suggests that the superior lip may be influenced by the vertical lengthening of the lips due to gravity, without any support from the inferior lip. To clarify this, the superior lip shape should be examined at rest and in dorsal position. Second, lengthening of the superior lip may be related to growth or aging [18]. Several previous reports have shown that growth and aging of the superior lip are related to lip thinning.

In general, orthodontists determine the inferior lip positions, and thus the mandibular incisor positions, to simulate the profiles, rather than relying on the superior lips. Our results (i.e., a strong correlation in the inferior lip) indicate that this traditional method (i.e., the inferior lip is the first determinant in deciding the treatment plan) is a reasonable approach because the inferior lip has fewer errors than the superior lip.

The present study has several limitations. First, it was a retrospective study. This can limit the quality and completeness of the data, and there may be missing data or inaccurate records. Second, this study only included patients who underwent orthodontic treatment with premolar extraction, which may not be representative of all orthodontic patients. Therefore, these results may not be generalizable to other treatment modalities or patient populations. Third, this study relied on cephalometric measurements, which may not accurately capture all aspects of facial soft tissue changes. Other imaging methods such as three-dimensional imaging or photography can provide a more comprehensive assessment of facial changes. Fourth, this study employed relatively uniform patients, namely one sex (female) and one ethnic group (Asian), which could possibly influence the results. Lastly, we included patients aged > 12 years in our study owing to the limited number of cases, and our findings indicate growth. Figure 1 shows a visual depiction of the mandibular growth across all groups. Although we acknowledge this limitation, we consider these results significant in clinical settings, given that orthodontic treatment predominantly occurs during the growth phase. Therefore, future studies should examine the effects of these factors.

## Conclusions

Changes in incisor inclination following orthodontic treatment were found to influence post-treatment soft tissue facial configurations. The mode of facial form change differed depending on the pre-treatment naso-lip-chin profile pattern. Repositioning of the superior and inferior lips in the four premolar extraction cases was predicted with moderate to high accuracy using regression models in the profile patterns that were determined using AI in the present report.

### Electronic supplementary material

Below is the link to the electronic supplementary material.

Supplementary Material 1

## Data Availability

The data produced and analyzed in this study are available from the corresponding author upon reasonable request.
