# Supplementary material for: Facial soft-tissue shape changes after fixed edgewise treatment with premolar extraction in individual artificial-intelligence-classified facial profile patterns
Source: BMC Oral Health. 2024 Jun 27;24:740. doi: 10.1186/s12903-024-04512-2 (PMC11209978; doi:10.1186/s12903-024-04512-2)
Supplement: Supplementary file 1 — Supplementary Material 1 [file 12903_2024_4512_MOESM1_ESM.docx]

**Supplementary Figures**

**
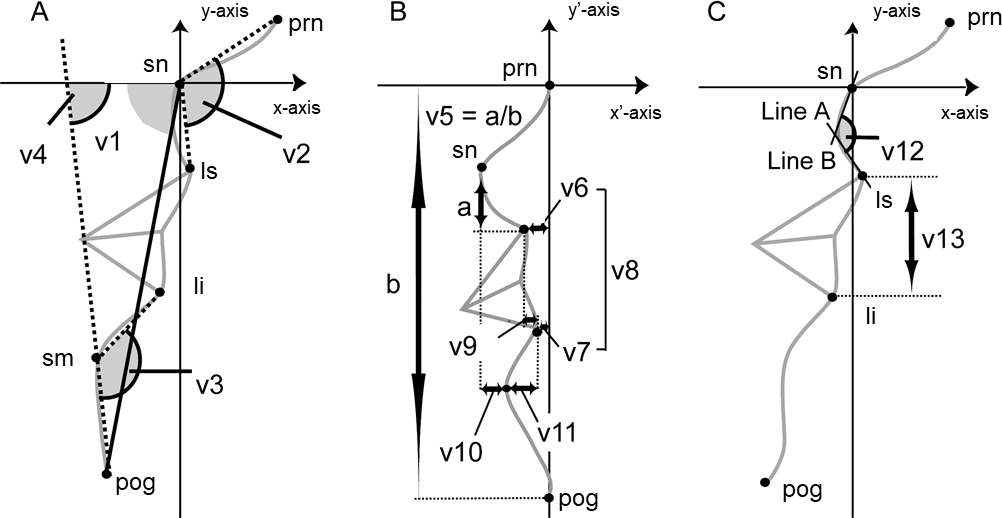
**

**Supplementary Figure 1:** Measurements for the nose-lip-chin profile ^1^. A: The x-axis was defined as the line that passes through the origin (subnasale [*sn*]) and is parallel to the Frankfort Horizontal (FH) plane. B: Pronasale [*prn*] was defined as the origin, the y′-axis as the line connecting prn and pogonion [*pog*]. C: Sn was defined as the origin, the x-axis as the line through the origin and parallel to the FH plane. Lines A and B are 1st-order polynomial approximations generated from the extracted contour data (for Line A, the data were extracted from *sn* to the midpoint of *sn* and labiale superius [*ls*]; for Line B, the data were extracted from *ls* to the midpoint of *sn* and *ls*). v1 designates the angle formed by the *sn-pog* line and the x-axis; v2, the angle formed by the *prn-sn* line and *sn-ls* line; v3, the angle formed by the *li*-submentale [*sm*] line and *sm-pog* line, the labio-mental angle; v4, the angle formed by the *sm-pog* line and the x-axis; v5, the value of [the difference between the y′-coordinate values of *sn* and *ls*]/[the difference between the y′-coordinate values of *prn* and *pog*]; v6, the x’-coordinate value of *ls*, the sagittal position of the superior lip vermilion, with a positive value indicating protrusion of the superior lip relative to the line connecting *prn* and *pog*; v7, the y′-coordinate value of *li*, with a positive value indicating protrusion of the inferior lip relative to the line connecting *prn* and *pog*; v8, the value of (v6 + v7); v9, the value of (v7 – v6); v10, the difference between the x′-coordinate values of *sn* and *sm*; v11, the difference between the x′-coordinate values of *sm* and *li*; v12, the angle formed by the approximated lines A and B, where Line A was defined as an approximated line between *sn* and the midpoint of *sn* and *ls* and Line B as an approximated line between the midpoint of *sn* and *ls* and v13, the difference between the y-coordinate values of *ls* and *li*.


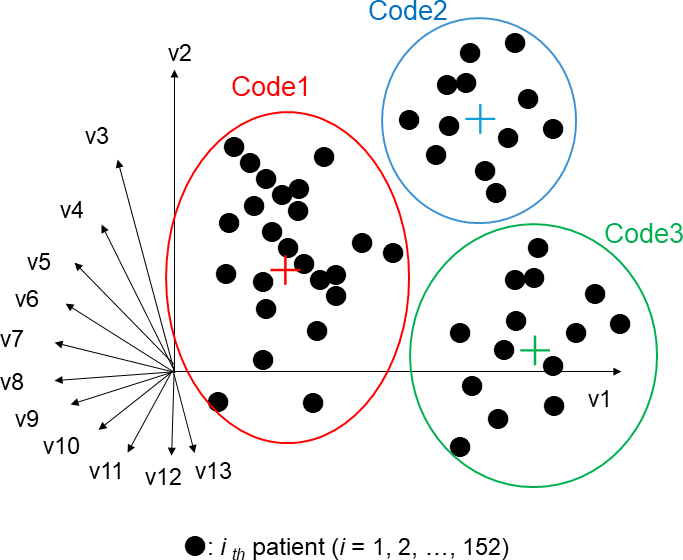


**Supplementary Figure 2:** Schematic illustration of an AI classifier (the vector quantization) in a 13-dimensional vector space. v1, v2, …, v13 indicates 13 vector elements consisting of a 13-dimensional vector (13 variables representing a face, see Supplementary Figure 1). In this figure, each patient was expressed as ● in 13-dimensional vector spaces. In the present study, calculations were made to classify the subjects represented as vectors (variable sets) into 2, 3, 4, 5, 6, 7, and 8 subcategories (code sets) (i.e., 2–8 code vectors). Here, we give the computation procedure by exemplifying classification into 3 code sets.

Vector quantization was performed as follows:

Step 1: Each facial profile was expressed by 13 vector elements (*vj*, j =1, 2, …, 13 as described in Supplementary Fig. 1), thus generating a 13-dimensional vector $\overset{\to}{\boldsymbol{V}}$.

Step 2: The initial 3 code vectors were determined from the 152 patients (a collection of 152 vectorized datasets, each vectorized dataset had 13 vector elements). These 3 initial code vectors were arbitrarily set according to the criterion that they were located farthest from each other when compared with the remaining combinations of interpoint distances and termed as initial code vectors 1, 2, and 3.

Step 3: Distances between an input vector $\overset{\to}{\boldsymbol{V}}$ and the 3 initial code vectors $\overset{\to}{\boldsymbol{C}}$ were computed, thus giving each patient 3 distances for each of the 3 initial code vectors. Here, the distance **D** between an input vector $\overset{\to}{\boldsymbol{V}}$ and the quantized code vector $\overset{\to}{\boldsymbol{C}}$ is expressed as:

$$\boldsymbol{D}\left( \overset{\to}{\boldsymbol{V}} ,\overset{\to}{\boldsymbol{C}} \right)= \sum_{j=1}^{13} \sqrt{{(vj-cj)}^{2}}$$

where *vj* represents the vector elements of an input vector $\overset{\to}{\boldsymbol{V}}$ and cj designates the vector elements of code vector $\overset{\to}{\boldsymbol{C}}$, where j takes values from 1 to 13.

Step 4: Each patient was categorized into a subcategory that corresponded to the initial code vector that showed the shortest distance from the vectors.

Step 5: The geometric centers (center of gravity) were calculated for each subcategory. The geometric centers Code 1, Code 2, and Code 3 for each of the 3 subsets thus obtained are assumed to be a new set of code vectors.

Step 6: The distances between each sample point and Code 1, Code 2, and Code 3 were computed to provide 3 new subsets. The calculation was repeated until the average deviation showed the minimum decremental rate when the optimal number of code sets was determined. Here, the average deviation designates the closeness of a vector that represents a facial form to the code vector to which the facial form belongs. In other words, the smaller the average deviation value, the higher the classification accuracy. The smaller the average deviation value, the closer the distance between the vector and its corresponding code vector. In short:

$$Average deviation= \frac{\sum_{i=1}^{153} \frac{\sum_{j=1}^{13} \sqrt{{(vij-cj)}^{2}}}{13}}{152}$$

where *vij* represents the jth vector element of the ith patient, and cj designates the vector element of the jth vector element of the corresponding code vector.


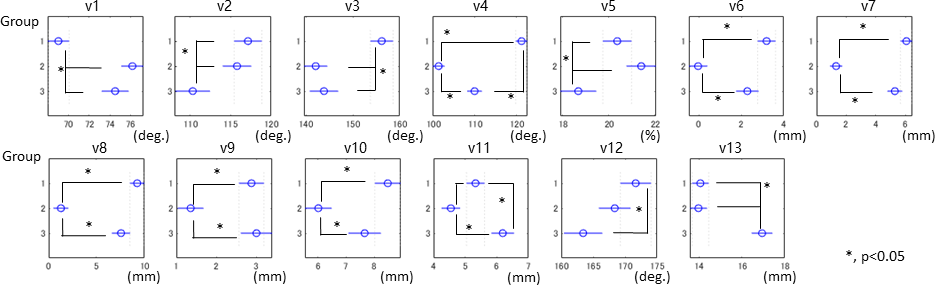


**Supplementary Figure 3 :** Inter-group comparisons of pretreatment mean values determined for the feature variables v1, v2,…, v13 that represented the naso-lip-chin profiles. *<0.05; ANOVA. Blue error bars represent Scheffé comparison intervals.

**
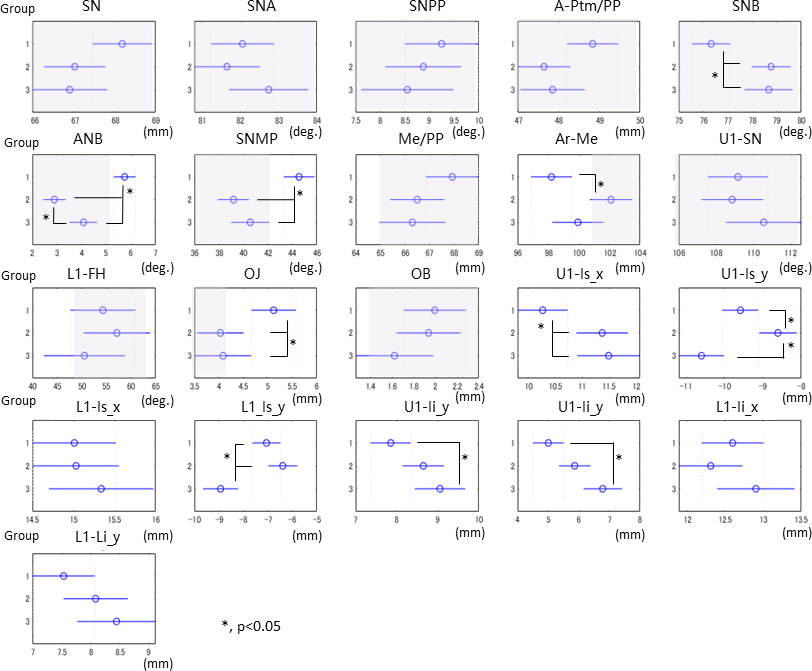
**

**Supplementary Figure 4:** Inter-group comparisons of pretreatment mean values in cephalometric measurements. *<0.05; ANOVA. Error bars represent Scheffé comparison intervals: nonoverlap between the bars of any two groups indicates that the hypothesis of no difference between the two was rejected at the P<0.05 level. Circle points represent estimated means. The transparent gray area indicates the normative range for SN, SNA, SNPP, A-Ptm/PP, SNB, ANB, SNMP, ME/PP, Ar-Me, U1-SN, L1-FH, OJ, and OB.

**REFERENCES**

1. Tanikawa C, Takada K. Objective classification of nose-lip-chin profiles and their relation to dentoskeletal traits. *Orthod Craniofac Res.* 2014;17(4):226-238.
